# Early fluid resuscitation with hyperoncotic hydroxyethyl starch 200/0.5 (10%) in severe burn injury

**DOI:** 10.1186/cc9086

**Published:** 2010-06-28

**Authors:** Markus Béchir, Milo A Puhan, Simona B Neff, Merlin Guggenheim, Volker Wedler, John F Stover, Reto Stocker, Thomas A Neff

**Affiliations:** 1 Division of Surgical Intensive Care, University Hospital of Zurich, Raemistrasse 100, Zurich, 8091, Switzerland; 2 Horten Centre for patient-oriented research, University Hospital of Zurich, Bolleystrasse 40, Zurich, 8091, Switzerland; 3 Department of Anaesthesiology, University Hospital of Zurich, Raemistrasse 100, Zurich, 8091, Switzerland; 4 Department of Reconstructive Surgery, University Hospital of Zurich, Raemistrasse 100, Zurich, 8091, Switzerland

## Abstract

**Introduction:**

Despite large experience in the management of severe burn injury, there are still controversies regarding the best type of fluid resuscitation, especially during the first 24 hours after the trauma. Therefore, our study addressed the question whether hyperoncotic hydroxyethyl starch (HES) 200/0.5 (10%) administered in combination with crystalloids within the first 24 hours after injury is as effective as 'crystalloids only' in severe burn injury patients.

**Methods:**

30 consecutive patients were enrolled to this prospective interventional open label study and assigned either to a traditional 'crystalloids only' or to a 'HES 200/0.5 (10%)' volume resuscitation protocol. Total amount of fluid administration, complications such as pulmonary failure, abdominal compartment syndrome, sepsis, renal failure and overall mortality were assessed. Cox proportional hazard regression analysis was performed for binary outcomes and adjustment for potential confounders was done in the multivariate regression models. For continuous outcome parameters multiple linear regression analysis was used.

**Results:**

Group differences between patients receiving crystalloids only or HES 200/0.5 (10%) were not statistically significant. However, a large effect towards increased overall mortality (adjusted hazard ratio 7.12; *P *= 0.16) in the HES 200/0.5 (10%) group as compared to the crystalloids only group (43.8% versus 14.3%) was present. Similarly, the incidence of renal failure was 25.0% in the HES 200/0.5 (10%) group versus 7.1% in the crystalloid only group (adjusted hazard ratio 6.16; *P *= 0.42).

**Conclusions:**

This small study indicates that the application of hyperoncotic HES 200/0.5 (10%) within the first 24 hours after severe burn injury may be associated with fatal outcome and should therefore be used with caution.

**Trial registration:**

NCT01120730.

## Introduction

In the VISEP (efficacy of volume substitution and insulin therapy in severe sepsis) study the application of hydroxyethyl starch (HES) 200/0.5 (10%) showed an increased incidence of renal failure in ICU patients, which was clearly dose-dependent. In fact the manufacturer's recommended dose of 20 ml/kg was exceeded in almost 60% of cases. The authors concluded that fluid resuscitation with HES 200/0.5 (10%) is harmful to patients with severe sepsis, because it leads to renal impairment and, at high doses, affects long-term survival. HES solutions should therefore be avoided in severe sepsis [1]. After publication of the VISEP trial there is an ongoing debate about fluid resuscitation, the role of crystalloids and colloids in the critically ill patient, the safety of HES, and even about the design of the VISEP study [2,3]. In this context and for ethical reasons (avoiding further harm to severe burn victims) we analyzed the results of this open-label interventional study, performed some years ago at our institution, to contribute to this important discussion.

Despite much experience in the management of severe burn trauma patients, controversies regarding the best type of fluid resuscitation, especially within the first 24 hours after trauma, are still going on.

In the early period after a severe burn, many pathophysiological changes take place. Systemic inflammation leads by release of different mediators such as leukotrienes, prostaglandins and particularly histamine, in combination with complement activation products to a massive capillary leak [4,5]. Intravascular molecules leak into the extravascular space, causing hypovolemia and shock [6]. Changes in capillary membrane permeability also produce electrolytic alteration with intracellular sodium accumulation with consecutive cellular swelling [7]. Tissue edema normally occurs within a few hours. Leakage of plasma proteins into the extravascular space contributes in a large extent to edema formation. The capillary leak is believed to stop between 8 and 24 hours after trauma, but data varys [4,8].

There is strong evidence that starting fluid resuscitation early improves clinical outcome in patient with severe burn injury [9], but there is no consensus about which kind of fluids would be the optimal treatment. In order to increase plasma osmolarity and thus reduce fluid losses into the extravascular space, some authors propose to add hypertonic solutions (e.g. hypertonic saline) in fluid resuscitation in these patients [10,11]. Fluid resuscitation in particular with excessive amounts of crystalloids in severe burn victims may lead to edema formation and thus contribute to respiratory failure, acute respiratory distress syndrome (ARDS) and/or abdominal compartment syndrome (ACS) [12]. ACS has a high impact on mortality in such patients and in one study 22 out of 25 patients died [13]. One of the treatment options for patients with ACS might be surgical abdominal decompression [14]. The main controversy about fluid resuscitation in severe burns is about the use or the avoidance of colloids, which solution to use and, last but not least, when to begin with the administration of colloids [15]. Among the available colloids, albumin and fresh frozen plasma (FFP) are mainly used. The Cochrane Injuries Group presented a relative risk of death after albumin administration of 2.4 in a metaanalysis [16]. Nevertheless, the infusion of albumin is very common in fluid management of severe burns [17,18].

The aim to reduce pulmonary complications (i.e. ARDS) and ACS by volume overload raised the issue of the application of colloids such as hyperoncotic HES. Colloids given after 24 hours in addition to crystalloids the extravascular lung water index did not increase [19]. The major concern about hyperoncotic HES 200/0.5 (10%) administration consists of its negative effects on renal function leading to renal failure and renal replacement therapy (RRT) [1]. Hyperoncotic HES used as a plasma-volume expander in brain-dead kidney donors has been shown to induce osmotic-nephrosis-like lesions and immediately impaired renal function in kidney-transplant recipients [20].

There is no evidence of whether the application of hyperoncotic HES 200/0.5 (10%) within the first 24 hours would improve or deteriorate the outcome in patients with severe burn injuries. Expert opinions of burn specialists consist of strictly avoiding colloids such as HES during the first 24 hours [21]. This restriction is based on reports from the early 1970 s expressing the fear of overloading the interstitial compartment with colloids due to increased capillary leakage in the early stage of trauma, which later might have negative effects on wound healing after surgical treatment [22,23].

Therefore, in our study we also addressed the question of whether the administration of hyperoncotic HES 200/0.5 (10%) within the first 24 hours in combination with crystalloids is as effective as crystalloids only in reducing the total amount of infused fluids and therefore might reduce complications such as pulmonary failure or abdominal compartment syndrome. Furthermore, we addressed overall mortality, the incidence of renal failure and whether surgical treatment could be started within the first three days after trauma.

## Materials and methods

The local ethical committee re-approved the analysis and protocol of the study in 2007 and waived the need for additional written informed consent for this data analysis 10 years after the study. Data were collected and analyzed from 30 consecutive patients with severe burns (> 20% body surface area) who were admitted to the burn unit of the University Hospital of Zurich, Switzerland, from August 1997 to July 1999. Patients were assigned in a prospective interventional open label study design either to a traditional crystalloids only ('Baxter group') resuscitation protocol (Baxter formula 4 ml crystalloids/kg/% deep burned body surface area) in the first 24 hours or to a new approach 'HES 200/0.5 (10%) group' with colloids and crystalloids (2 ml crystalloids/kg/% deep burned body surface area plus 0.5 ml HES 200/0.5 (10%)/kg/% deep burned body surface area). The crystalloid given was lactated Ringer's solution (LR). Topical treatment of burn wounds was standardized in all patients using silver sulfadiazine.

The patient characteristics are shown in Table 1. Groups were not well balanced for age. Therefore, we used multivariable regression models (Cox proportional hazard regression for time to event data and linear regression for continuous outcomes) with outcomes as the independent variable and group as the dependent variable while adjusting for potential confounders (age, gender percent burn, acute physiology and chronic health evaluation (APACHE) II, baseline creatinine).

**Table 1:** Participant characteristics

	Baxter group (n = 14)	HES group (n = 16)	*P *value
**Age **(years)	35.9 ± 14.2*	49.4 ± 22.0	0.06
**Number of male participients **(%)	11 (78.6)	13 (81.3)	0.86
**Weight **(Kg)	75.3 ± 11.3	72.7 ± 15.2	0.60
**APACHE II score **(points)	8.4 ± 5.8	9.1 ± 2.2	0.65
**Creatinine baseline **(μmol/l)	74.6 ± 9.8	82.7 ± 19.7	0.18
**2^nd ^+ 3^rd ^degree burn **(%)	37.4 ± 14.2	40.0 ± 13.8	0.62
**Burn type **(number of patients)			0.17
- fire	12	11	
- fire/explosion	1	1	
- steam/scalding liquids	0	4	
- electrical/chemical	0	0	
- flash injury	1	0	
**Number of patients with inhalation injury **(%)	2 (14.2)	3 (21.4)	0.51
**Time from burn to iv-start **(minutes)	100 ± 153	59 ± 38	0.44
**Time from burn to hospital admission **(minutes)	175 ± 173	141 ± 81	0.90
**Time to first surgery **(days)	5 ± 4	4 ± 3	0.87

* Values are means ± standard deviation unless stated differently. APACHE, acute physiology and chronic health evaluation; HES, hydroxyethyl starch.

### Study protocol

After admission to our hospital patients were assigned either to the traditional crystalloids only (Baxter group) or to the experimental resuscitation regimen (HES 200/0.5 (10%) group). The estimated amounts of fluids were calculated according to the above mentioned formulas. Except for the different fluid management, all patients were treated and monitored in the same manner according to the same target variables (urinary output ≥1.0 ml/kg/hour, mean arterial pressure ≥65 mmHg, hematocrit 35% to 45%, and serum lactate ≤2.0 mmol/l). If target values could not be reached with volume therapy alone, norepinephrine infusion was added. Blood glucose levels were kept between 6 to 12 mmol/l. Besides the ICU standard monitoring we assessed the administered amount of fluids, creatinine clearance (Cockroft formula) every 24 hours for the first three days, urinary output, the Horovitz-Index after 72 hours, ventilator days and the incidence of ARDS. We analysed hematocrit, white blood cells (WBC), C-reactive protein (CRP), glutamat-oxalacetat-transaminase and glutamat-pyruvat-transaminase (GPT) at days one, two, three and seven. Furthermore, we assessed ICU and hospital days, the incidence of sepsis, overall mortality, and the beginning of surgical procedures within the first three days after trauma.

### Statistical analysis

Binary data are presented as proportions and continuous variables as mean ± standard deviation. For binary outcomes we used a Cox proportional hazard regression with the event (e.g. mortality) as dependent variable and fluid resuscitation therapy (HES or Baxter) as independent variable. As this was not a randomized trial we adjusted for potential confounders (age, gender, percent burn, APACHE II and baseline creatinine) in the multivariate regression models. For continuous outcomes (e.g. creatinine clearance) we used a multiple linear regression analysis with the same independent variables as described above. For comparison of the blood markers we used the method of analysis of variance for repeated measurements (adjusted for potential confounders age, gender, percent burn, APACHE II and baseline creatinine). We conducted all analyses using SPSS for Windows (version 12.0.1, SPSS Inc, Chicago, Illinois, USA).

## Results

Thirty patients were included in the study, 14 to the crystalloids only group and 16 to the HES 200/0.5 (10%) group. The patient characteristics including degree of burn are shown in Table 1.

### Was there a difference in the total amount of fluids?

The estimated amount of fluids for the first 24 hours was 11,150 (± 4115) ml LR in the crystalloids only group versus 7,082 (± 5142) ml LR and 1,409 (± 642) ml HES in the HES 200/0.5 (10%) group. The effectively given amounts of fluids in the two groups over the days one to three are shown in Table 2. Approximately 1.5-fold of the initially calculated amount of fluid replacement was given to both groups. There was a protocol violation as four patients in the crystalloids only group were treated with 1325 (± 538) ml HES during the operating procedures at days two and three and one patient of the HES 200/0.5 (10%) group received 200 ml of albumin during operating procedures.

**Table 2:** The intervention protocol

	Baxter group (n = 14)	HES group (n = 16)
**Fluid volumes calculated for first 24 hours**		
Lactated Ringer's Solution (ml)	11,150 ± 4,115*	7,082 ± 5,142
HES (ml)	0	1,409 ± 642
**Fluid volumes given**		
Lactated Ringer's Solution (ml)		
After 24 hours	18,667 ± 9,438	12,692 ± 4,785
After 48 hours	22,220 ± 11,340	16,122 ± 5,307
After 72 hours	24,903 ± 13,093	18,951 ± 7,113
HES (ml)		
After 24 hours	1 patient: 200	3,431 ± 1,674
After 48 hours	2 patients: 200 each	4,966 ± 2,461
After 72 hours	4 patients: 1,325 ± 538	6,094 ± 3,359
Albumin (ml)		
After 24 hours	9 patients: 136 ± 70	0
After 48 hours	13 patients: 438 ± 119	0
After 72 hours	11 patients: 627 ± 205	1 patient: 200

* Values are means ± standard deviation unless stated differently. HES, hydroxyethyl starch.

Overall, the addition of colloids and crystalloids reveals that there was no difference in the total amount of fluids given between the groups.

### Was there an influence on mortality or renal failure?

**Figure 1:**
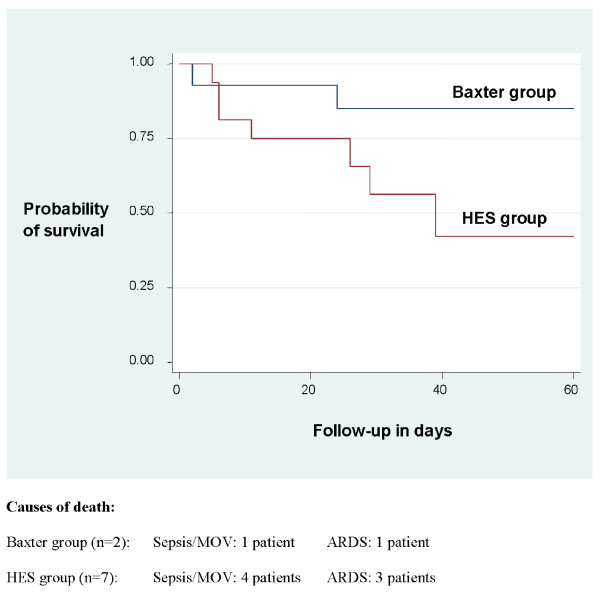
**Kaplan-Meier survival curve and causes of death**. Large effect towards a seven-fold higher mortality in the HES group (adjusted hazard ratio 7.12; *P *= 0.16). ARDS, acute respiratory distress syndrome; HES, hydroxyethyl starch.

No statistically significant differences were found between the HES 200/0.5 (10%) and crystalloids only groups. However, a large effect towards increased mortality (hazard ratio 7.12) and renal failure leading to continuous RRT (CRRT; hazard ratio 6.16) was detected for HES (Table 3 and Figure 1). Looking at single patients developing renal failure, CRRT was started on days 8 to 19 postinjury. Renal parameters such as hourly urinary output after 72 hours and daily creatinine clearance (cockroft formula) were not significantly different.

**Table 3:** Outcomes

Outcome			Baxter group (n = 14)	HES group (n = 16)	Adjusted hazard ratio or mean difference (95% CI, *P *value)^#^
**Mortality**	**Overall mortality **(%)		2 (14.3)	7 (43.8)	Hazard ratio: 7.12 (0.45-112.7, *P *= 0.16)

**Renal parameters**	**Continuous renal replacement therapy **(%)		1 (7.1)	4 (25.0)	Hazard ratio: 6.16 (0.07-505.7, *P *= 0.42)
	**Creatinine clearance**	24 hours	125.9 (20.1)	102.5 (38.8)	Mean difference: -6.5 (-26.8 to 13.7, *P *= 0.51)
		48 hours	125.9 (25.6)	95.6 (40.3)	Mean difference: -14.7 (-37.8 to 8.4, *P *= 0.20
		72 hours	124.2 (33.4)	97.4 (47.2)	Mean difference: -4.8 (-28.3 to 18.6, *P *= 0.67)
	**Urinary output**	72 hours	165.1 (53.1)	135.8 (59.3)	Mean difference: -9.7 (-56.6 to 37.2, *P *= 0.67)

**Pulmonary parameters**	**ARDS **(%)		4 (28.6)	3 (18.8)	Hazard ratio: 1.26 (0.14-11.35, *P *= 0.84)
	**Ventilator days**		7.4 (11.0)	12.3 (19.7)	Mean difference: -2.4 (-12.9 to 8.2, *P *= 0.65)
	**Horowitz**	72 hours	237.2 (98.3)	225.4 (85.7)	Mean difference: 17.5 (-51.7 to 86.6, *P *= 0.61)

**Surgical parameters**	**Beginning of surgical treatment within first 3 days **(%)		10 (71.4)	9 (56.3)	Hazard ratio: 2.00 (0.58-6.93, *P *= 0.27)
	**Time to complete surgical coverage **(days)		23.0 (15.7)	31.9 (30.8)	Mean difference: 1.7 (-25.2 to 28.6, *P *= 0.89)

**Other parameters**	**Sepsis **(%)		5 (35.7)	6 (37.5)	Hazard ratio: 0.95 (0.16-5.56, *P *= 0.95)
	**Hospital days**		32.4 (16.1)	28.6 (20.7)	Mean difference: -6.7 (-22.9 to 9.6, *P *= 0.40)
	**ICU days**		27.0 (14.1)	23.6 (17.4)	Mean difference: -7.6 (-21.1 to 5.8, *P *= 0.25)

^# ^Multivariable logistic regression analysis or linear regression analysis with outcome as independent and groups as dependent variable with adjustment for age, gender percent burn, acute physiology and chronic health evaluation (APACHE) II, baseline creatinine. ARDS, adult respiratory distress syndrome; CI, confidence interval; HES, hydroxyethyl starch.

In conclusion, the application of HES 200/0.5 (10%) may have a negative impact on mortality and may promote renal failure.

### Was there a reduction of complications?

There were no differences between the groups in the incidence of ARDS, ventilator days and the Horovitz quotient after 72 hours. ACS occurred in none of the patients. The incidence of sepsis was the same in both groups. The adjusted length of hospital and ICU stay was not different between the groups (Table 3).

Taken together, there was no difference in complications between the groups.

### Was the surgical procedure disturbed with the application of HES?

Surgical treatment of the groups did not differ; neither the timepoint of the first surgical intervention (day three surgery) nor completion of surgical coverage of the burned body surface areas was delayed (Table 3).

The application of colloidal HES did not influence timing of surgical procedures.

### Was there a difference in blood markers between the groups?

**Figure 2:**
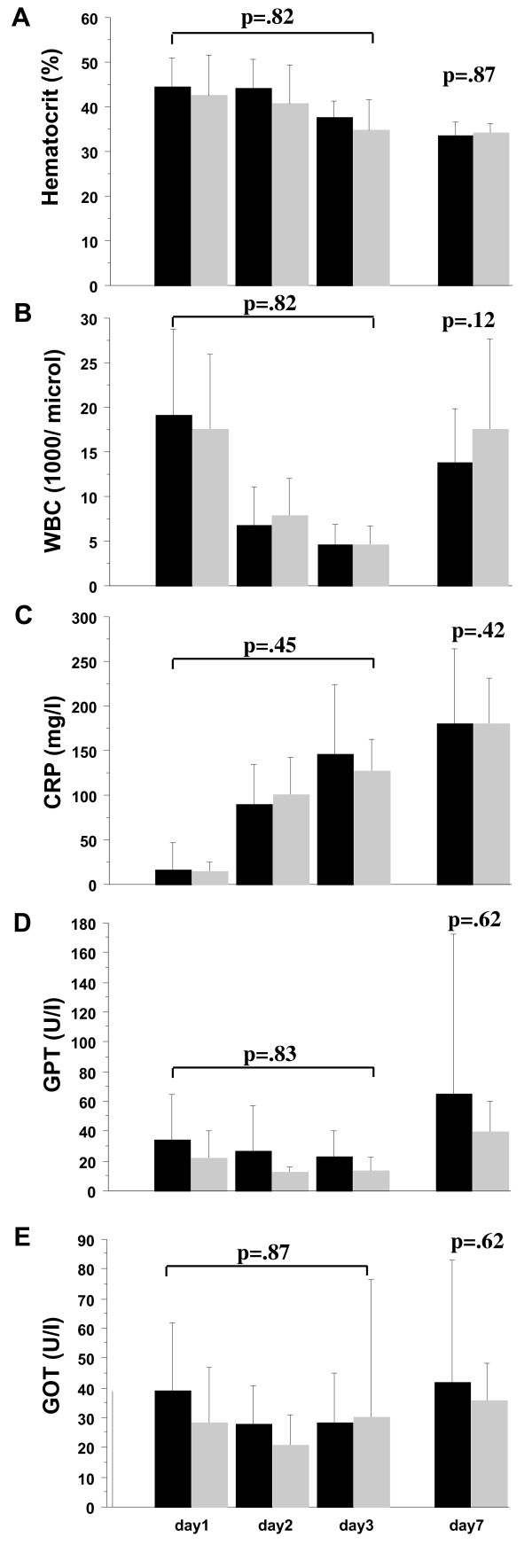
**Bloodmarkers at days one, two, three and seven**. **(a) **Hematocrit, **(b) **white blood count, **(c) **C-reactive protein, **(d) **glutamat-pyruvat-transaminase (GPT) and **(e) **glutamat-oxalacetat-transaminase (GOT). Baxter group black bars, hydroxyethyl starch (HES) group grey bars. Plots show mean ± standard deviation, analysis of variance for repeated measurements. There were no differences between the groups.

Hematocrit, WBC, CRP and GPT did not differ between groups during the first seven days of the study (Figure 2).

We could not discriminate any different pattern of inflammation or hematocrit between the two groups.

## Discussion

Similarly to the VISEP trial in septic patients our study also showed that the application of HES 200/0.5 (10%) in a population of severely burned patients may be associated with fatal outcome in comparison to traditional fluid resuscitation with crystalloids only.

A potential explanation for the large effect of possibly increased mortality in the HES 200/0.5 (10%) group might be the detected six-fold higher rate of renal failure needing CRRT. There is strong evidence for increased mortality after renal failure in ICU patients [24]. In a large multicenter trial an overall mortality of 60.3% in ICU patients with acute renal failure was found [25].

About 33 to 66% of administered hyperoncotic HES is excreted in the urine in the first 24 hours after infusion [26]. Some hyperoncotic HES remains in circulation for a long time and a substantial proportion accumulates in various tissues, including the kidneys. In dogs, hyperoncotic HES deposition was demonstrated by histopathology in intravascular and interstitial spaces, parenchymal liver cells, proximal renal tubular cells, and phagocytes in liver, spleen, lymph nodes and other organs [27]. There are many case studies describing acute deterioration of pre-existing renal impairment after the administration of hyperoncotic HES [28,29]. Interestingly, in the HES group both of the two patients with the highest baseline creatinine level (> 110 mmol/l) died. Renal biopsies of such patients often show osmotic nephrosis-like lesions [30].

There are outcome studies after hyperoncotic HES administration, but none deal with severely burned patients. In a multicenter randomized trial of 129 patients with severe sepsis or septic shock, hyperoncotic HES administration was an independent risk factor for acute renal failure, with an adjusted odds ratio of 2.5 [31]. Renal failure after hyperoncotic HES hemodilution was also described in patients after cardiac surgery [32,33], abdominal surgery [34], and renal transplantation [35].

Our results are clearly in line with the VISEP trial. A hyperoncotic 10% HES 200/0.5 was administered in both studies, and the manufacturers maximal amount of 20 ml/kg/24 hours of HES was also exceeded in 11 of 16 (68%) of our patients. Thus, we must emphasize that the overtreatment with 'old' hyperoncotic HES 200/0.5 (10%) may at least in part have been responsible for the possible negative effects of HES on morbidity and mortality in our study population.

Furthermore, we could not discriminate a reduction of ARDS. In another studies the infusion of additional albumin in severe burns was able to reduce the total amount of fluids during resuscitation. Interestingly, extravascular lung water and capillary permeability is rarely elevated after such treatment [36]. In one study comparing colloids with crystalloids more saline than colloid solutions was infused, cardiac output increased more in the colloid groups, and HES seemed to ameliorate increased pulmonary permeability [37]. In contrast, our data could not demonstrate that the administration of HES 200/0.5 (10%) could improve pulmonary function.

The incidence of sepsis in ICU patients is well documented and analyzed. It affects about 40% of ICU admissions, severe sepsis occurs in about 30%, and septic shock in 15% [38]. The incidence of sepsis in our study did not differ between the groups. About one-third in each group developed sepsis, which is in line with the literature. Also, we could not find any significant differences in inflammation markers (WBC, CRP), as well as in length of hospital stay and ICU days.

In a rabbit model, intraoperative profound hemodilution with hyperoncotic HES did not interfere with small-intestinal wound healing as long as postoperative haemoglobin levels were maintained above 10 g/100 ml [39]. Another study showed that the infusion of hyperoncotic HES and saline reduced acute microvascular deteriorations, trauma-induced inflammatory response and tissue edema in rats [40]. In our study, there was no difference regarding either the beginning of the surgical procedures within the first three days after trauma or with respect to the time to complete coverage of the burned body surface areas. Therefore, we cannot support the concerns about impaired wound healing after the application of colloids.

### Study limitations

This study was not a randomized controlled trial with blinding of patients and physicians. Although we adjusted for potential confounders there might be residual confounding in our estimates. Also, the sample size was small leading to imprecise estimates. For example, although we observed a higher mortality rate in patients with HES, the difference was not significant. Nevertheless, the detection of a seven-fold higher mortality in this prospective interventional study with such a small sample size is an impressive finding and has to be interpreted very carefully. A similar statistical situation was the CLARICOR (effect of clarithromycin on mortality and morbidity in patients with ischemic heart disease) study, in which the analysis of clarithromycin in patients with stable coronary heart disease showed also a non-significant hazard ratio [41], but the Food and Drug Administration reacted on that study with a warning notice. In our study the non-significance might be a consequence of the small sample size. Hence, in a 'dark' field without data from randomized controlled trials and with regard to the ongoing debate after the VISEP study these results are important, especially as no randomized controlled trial data are available.

There was some protocol violation as four patients of the Baxter group received HES during operating procedures, but not during the first 24 hours after trauma. The amount of HES was relatively small compared with the whole administered fluids. This fact theoretically would have reduced the difference between the groups, because HES could have deteriorated the patients in the Baxter group.

As mentioned above there was a trend to a difference in age between the groups, which was statistically appropriately adjusted. Therefore, a hazard ratio of 7.12 is a massive difference in mortality and hardly explainable by the difference of baseline characteristics only. However, if a randomized controlled trial was designed based on the mortality rates we observed (44% in the HES group and 14% in the Baxter group) and to detect a significant difference at a significance level of 0.05 and a power of 80%, it would require 42 patients per group (without drop-outs).

## Conclusions

In summary, our study showed that the application of hyperoncotic HES 200/0.5 (10%) within the first 24 hours after severe burn injury may be associated with increased mortality and renal failure as compared with traditional fluid resuscitation with crystalloids only, but findings were not significant.

## Key messages

• There is some indication that HES 200/0.5 (10%) may be associated with increased mortality and renal failure in patients with severe burn injury, but findings are not significant.

• HES 200/0.5 (10%) should be used with caution in patients with severe burn injury.

• Successful surgery in burn injury is not affected by the application of HES 200/0.5 (10%).

## Abbreviations

ACS: abdominal compartment syndrome; APACHE: acute physiology and chronic health evaluation; ARDS: acute respiratory distress syndrome; CRP: C-reactive protein; CRRT: continuous renal replacement therapy; FFP: fresh frozen plasma; GPT: glutamat-pyruvat-transaminase; HES: hydroxyethyl starch; LR: lactated Ringer's solution; RRT: renal replacement therapy; WBC: white blood count.

## Competing interests

The authors declare that they have no competing interests.

## Authors' contributions

JFS, MG and VW collected the majority of the data and drafted parts of the manuscript. MP performed statistical analysis. SBN and RS helped analyzing and interpreting the data and drafted parts of the manuscript. MB and TAN led the project, collected parts of the data, performed additional statistical analysis and drafted parts of the manuscript.
